# Comparison of non-traditional biomarkers, and combinations of biomarkers, for vascular risk prediction in people with type 2 diabetes: The Edinburgh Type 2 Diabetes Study

**DOI:** 10.1016/j.atherosclerosis.2017.07.009

**Published:** 2017-09

**Authors:** Anna H. Price, Christopher J. Weir, Paul Welsh, Stela McLachlan, Mark W.J. Strachan, Naveed Sattar, Jackie F. Price

**Affiliations:** a Usher Institute of Population Health Sciences and Informatics, University of Edinburgh, Edinburgh, Scotland, UK; b Edinburgh Clinical Trials Unit, University of Edinburgh, Scotland, UK; c Glasgow Cardiovascular Research Centre, University of Glasgow, Glasgow, Scotland, UK; d Metabolic Unit, Western General Hospital, Edinburgh, Scotland, UK

**Keywords:** Cardiovascular disease, Type 2 diabetes, Biomarkers, Risk prediction, Epidemiology

## Abstract

**Background and aims:**

We aimed at comparing the impact of multiple non-traditional biomarkers (ankle brachial pressure index (ABI), N-terminal pro-brain natriuretic peptide (NT-proBNP), high sensitivity cardiac troponin (hs-cTnT), gamma-glutamyl transpeptidase (GGT) and four markers of systemic inflammation), both individually and in combination, on cardiovascular risk prediction, over and above traditional risk factors incorporated in the QRISK2 score, in older people with type 2 diabetes.

**Methods:**

We conducted a prospective study of 1066 men and women aged 60–75 years with type 2 diabetes mellitus, living in Lothian, Scotland.

**Results:**

After 8 years, 205 cardiovascular events occurred. Higher levels of hs-cTNT and NT-proBNP and lower ABI at baseline were associated with increased risk of CV events, independently of traditional risk factors (basic model). The C statistic of 0.722 (95% CI 0.681, 0.763) for the basic model increased on addition of individual biomarkers, most markedly for hs-cTnT (0.732; 0.690, 0.774)). Models including different combinations of biomarkers had even greater C statistics, with the highest for ABI, hs-cTnT and GGT combined (0.740; 0.699, 0.781).

**Conclusions:**

Individually, hs-cTnT appeared to be the most promising biomarker in terms of improving vascular risk prediction in people with type 2 diabetes, over and above traditional risk factors incorporated in the QRISK2 score. Combining several non-traditional biomarkers added further predictive value, and this approach merits further investigation when developing cost effective risk prediction tools for use in clinical practice.

## Highlights

• hs-cTnT is the most promising biomarker in terms of improving vascular risk prediction in people with type 2 diabetes.
• Combining biomarkers adds further predictive value.
• A number of biomarkers were included for comparison with each other within one study population.
• An unbiased approach to selecting biomarkers was taken.
• The basic model was based on a risk score currently recommended for use in type 2 diabetes for risk prediction in the UK.

## 1 Introduction

The risk of cardiovascular (CV) disease is increased two-fold in people with type 2 diabetes [1]. In the UK, the National Institute for Health and Care Excellence (NICE) clinical guidelines recommend the use of the QRISK2 score [2] to calculate 10-year CV risk; this score combines several traditional CV risk factors and has been validated in patients with and without type 2 diabetes [3]. Numerous CV risk scores have been recommended worldwide, but, in general, all current scores appear to perform inadequately in people with type 2 diabetes, either under- or over-estimating risk of CV events [4], [5], [6]. Although people with type 2 diabetes are routinely offered lifestyle advice and treatment with lipid-lowering agents after diagnosis, better risk stratification may allow targeted use of aggressive prevention strategies.

Increasing numbers of studies have suggested biomarkers, which might improve vascular risk prediction scores in the general populations, and, to a lesser extent, in diabetic study populations [7], [8], [9], [10], [11], [12]. However, such studies have tended to look at the addition of single risk factors over-and-above a small panel of traditional risk factors (or an established risk score based on such traditional risk factors). A direct comparison of the value of different non-traditional biomarkers has not been well evaluated within the setting of a single epidemiological study. Similarly, the value of different combinations of the most promising biomarkers has not been well studied.

The aim of the current research was to compare the addition of a number of different biomarkers to a vascular risk score based as closely as possible on that currently recommended for clinical use in people with diabetes in the UK (QRISK2), and to investigate the extent to which different combinations of these various biomarkers might improve prediction. The biomarkers selected included those identified in previous research, especially those which might be of particular importance in diabetes (such as inflammatory markers which are generally raised in people with diabetes and gamma GT as a measure of liver dysfunction which has been linked with both the high prevalence of fatty liver disease in diabetes and with cardiovascular disease). Since the overall aim was to provide results, which would be informative for potential application in a clinical setting, biomarkers selected were also restricted to those which can be relatively easily measured in a routine clinic setting, either by means of a blood test (N-terminal prohormone of brain natriuretic peptide (NT-proBNP), high-sensitivity cardiac troponin T (hs-cTnT), gamma-glutamyl transpeptidase (GGT) and markers of systemic inflammation such as C-reactive protein (CRP), interleukin-6 (IL-6), tumor necrosis factor alpha (TNF-α) and fibrinogen) or by means of a straightforward physical test (the ankle brachial pressure index (ABI).

## 2 Materials and methods

### 2.1 Study population

The Edinburgh Type 2 Diabetes Study (ET2DS) is a population-based, prospective cohort of 1066 men and women aged 60–75 years with established type 2 diabetes living in the Lothian region of Scotland, UK. In 2006/2007, participants were recruited at random from the Lothian Diabetes Register, a registry of almost all people with type 2 diabetes living in Lothian, resulting in a cohort largely representative of this target population [13]. Recruitment and data collection using questionnaires and physical examination at baseline research clinics (supplemented with data from the Information Services Division (ISD) of National Health Service Scotland on all medical and surgical discharge records from Scottish hospitals since 1981) have been described previously [14]. Subjects have been followed up over 8 years for development of CV events. All study participants gave informed consent and ethical approval was granted by the Lothian Medical Research Ethics Committee.

### 2.2 Assessment of traditional CV risk factors for the basic model

A range of traditional CV risk factors to be included in a basic risk prediction model were measured at baseline using methods described previously in detail [14]. Risk factors were chosen and defined to replicate variables included in QRISK2 as closely as possible, including age, sex, smoking, atrial fibrillation (AF), rheumatoid arthritis (RA), hypertension, chronic kidney disease (CKD), body mass index (BMI), systolic brachial blood pressure (sBP), total:high density lipoprotein (HDL) cholesterol and social status. Self-reported smoking history from questionnaires was categorised as: non-smoker, ex-smoker, <10 cigarettes/day, 10–19 cigarettes/day and 20 + cigarettes/day. RA was recorded from a combination of self-report and hospital discharge codes in the record linkage data from ISD. AF was recorded from self-reported use of digoxin, relevant hospital discharge codes or AF on the 12-lead ECG taken at the research clinic. Hypertension was defined as self-report of anti-hypertensive medication. CKD was defined as an estimated glomerular filtration rate (eGFR) < 60 ml/min on 2 of 3 consecutive measurements in the 12–24 months prior to baseline in routine biochemistry data extracted from the Lothian Diabetes Register to replicate ‘doctor diagnosis of CKD’ used in QRISK2. Social status was categorised using the Scottish Index of Multiple Deprivation (SIMD) based on post code [15].

### 2.3 Assessment of non-traditional biomarkers

Plasma from fasting venous blood samples taken at baseline was frozen for storage. Plasma NT-proBNP and hs-cTNT were subsequently measured using the Elecsys 2010 electrochemiluminescence method (Roche Diagnostics, Burgess Hill, UK), and calibrated using the manufacturer's reagents. The manufacturer's controls were used with limits of acceptability defined by the manufacturer. GGT was analysed using a Vitros Fusion chemistry system (Ortho Clinical Diagnostics, High Wycombe, UK) at the Western General Hospital, Edinburgh, UK. Assays for plasma TNF-α, IL-6, CRP and fibrinogen were carried out in the University Department of Medicine, Glasgow Royal Infirmary. TNF-α and IL-6 antigen levels were determined using high-sensitivity ELISA kits (R&D Systems, Oxon, UK). CRP was assayed using a high-sensitivity immunonephelometric assay. Fibrinogen assays were performed using stored plasma anticoagulated with trisodium citrate and the automated Clauss assay (MDA-180 coagulometer, Organon Teknika). ABI was measured as described previously as the ratio of systolic BP in the ankle to that in the arm [14]. Participants with a value > 1.4 (n = 17), indicative of medial arterial calcinosis rather than atherosclerosis, were subsequently omitted, in line with previous studies [29].

### 2.4 Assessment of CVD events

Using pre-defined criteria [13], data collected at baseline from questionnaires, ECG and hospital discharges were used to record prevalent CVD (MI, angina, transient ischaemic attack (TIA), stroke and coronary intervention). Data used to identify incident CV events over 8 years included ECGs plus self-reported and GP-reported events in questionnaires, all completed after 4 years, plus ISD record linkage for hospital discharge and death certificate data together with review of clinical case notes at both four and eight years. Criteria for fatal and non-fatal events were as follows. MI: ICD-10 code for new MI (I21—I23, I252) on discharge/death record, dated after baseline, confirmed by self-reported doctor diagnosis of MI, positive WHO chest pain questionnaire for MI, report of MI on GP questionnaire, new ECG changes or inspection of clinical notes. Angina [1]: ICD-10 code for angina (I20—I25) as primary diagnosis on hospital discharge record, dated after baseline, with no previous indication of angina; or [2] at least two of (a) self-reported doctor diagnosis of angina or new angina medication since baseline, (b) ECG codes for ischaemia that were not present at baseline and (c) positive WHO chest pain questionnaire. Fatal ischaemic heart disease (IHD): subject did not meet any of the criteria for fatal MI and had an ICD-10 code for IHD (I209, I249, I258, I259) as primary cause of death. Stroke [1]: ICD-10 code for stroke (I61, I63—I66, I679, I694) as primary diagnosis on discharge/death record, dated after baseline; or [2] self-report of stroke or non-primary ICD-10 discharge/death code for stroke dated after baseline, both confirmed on scrutiny of clinical notes. TIA [1]: ICD-10 code for TIA (G45, G659) as primary diagnosis on discharge record; or [2] self-report of stroke or non-primary ICD-10 discharge code for stroke or TIA dated after baseline, confirmed as TIA on scrutiny of clinical notes. Coronary intervention: OPCS operation code for coronary intervention (K40—K44, K49) on discharge record.

### 2.5 Statistical analysis

Distributions of ABI, NT-proBNP, hs-cTNT, Gamma-GT, TNF-α, CRP and IL-6 were skewed and therefore log-transformed in analyses. The Pearson correlation coefficient *r* and test of association were used to assess relationships between biomarkers. The four inflammation biomarkers (TNF-α, CRP, IL-6 and fibrinogen) were combined into one general inflammation factor using an unrotated principal components analysis. All four markers loaded quite strongly onto the first principal component (0.44–0.80), which explained 49% of the total variability, and this was used to calculate the general inflammation factor, *g*. An incident CV event was defined as the first CV event (fatal or non-fatal MI or stroke, fatal IHD, angina, TIA or coronary intervention) occurring after baseline.

In addition to components of the QRISK2 score (in the development of which subjects with prior CVD or taking statins were excluded) with the exception of family history of CV disease which was not available in the ET2DS, baseline CVD status and use of lipid lowering medication were also included to produce a basic model. The corresponding coefficients were estimated directly from ET2DS data. Binary logistic regression models were used to evaluate the relationships between each biomarker and CV events, chosen in favour of Cox regression to avoid having to make the proportional hazards assumption. The added predictive value of including each biomarker to the basic model was assessed using C statistics (model discrimination). The net reclassification index (NRI) was calculated, as well as the net reclassification (NR) separately for participants who experienced a CV event and those who did not, giving the change in the proportion of subjects correctly classified according to pre-specified CV risk categories (0–10%, 10–20% and >20%). Calibration was assessed using the Hosmer-Lemeshow test (*p*-value > 0.05 indicates good calibration). All subsets regression was used to compare all possible combinations of biomarkers and obtain the best five models, according to a pre-specified statistical criterion (Akaike's Information Criterion, AIC) which measures the relative quality of a model while penalising for increasing numbers of predictors). In addition, a model was fitted which included conventional CV risk factors and the full panel of biomarkers.

## 3 Results

### 3.1 Study characteristics at baseline and incident CV events

Due to low numbers of non-white participants (*n* = 17), all analyses were restricted to Caucasian participants (n = 1049; 515 women, 534 men). Mean age at baseline was 67.9 ± 2.4 years. Baseline prevalences of MI, angina, stroke, TIA and coronary intervention were 14.0% (*n* = 147), 27.8% (*n* = 292), 5.8% (*n* = 61), 2.9% (*n* = 30) and 10.1% (*n* = 106) respectively. Use of lipid lowering medication was reported by 896 subjects (85.4%). Full baseline characteristics of the study population are shown in Table 1. Correlations between biomarkers, shown in Table 2, were particularly strong for the inflammatory markers.

**Table 1:** Baseline characteristics of the ET2DS population.

Variable	
Age (years)	67.9 ± 4.2
Sex (female)	515 (49.1)
Lipid-lowering medication	896 (85.4)
Hypertension	858 (81.8)
Smoking status	
Non-smoker	411 (39.2)
Ex-smoker	491 (46.8)
Current smoker – light (<10 cigarettes/day)	31 (3.0)
Current smoker – moderate (10–19 cigarettes/day)	47 (4.5)
Current smoker – heavy (20 + cigarettes/day)	69 (6.6)
Atrial fibrillation	69 (6.6)
Chronic kidney disease	258 (24.6)
Rheumatoid arthritis	39 (3.7)
SIMD	
Quintile 1 (most deprived)	127 (12.1)
Quintile 2	205 (19.5)
Quintile 3	185 (17.6)
Quintile 4	192 (18.3)
Quintile 5 (least deprived)	340 (32.4)
BMI (kg/m^2^)	31.5 ± 5.7
sBP (mmHg)	133.3 ± 16.5
Total cholesterol: HDL cholesterol	3.5 ± 1.1
CVD at baseline^a^	
MI	147 (14.0)
Angina	292 (27.8)
Stroke	61 (5.8)
TIA	30 (2.9)
CI	106 (10.1)
ABI	1.0 (0.9, 1.1)
NT-proBNP (pg/ml)	76 (38, 172)
hs-cTnT (ng/L)	9.6 (6.9, 13.8)
GGT (U/L)	18 (11, 32)
TNF-α (pg/ml)	1.1 (0.7, 1.6)
IL-6 (pg/ml)	2.9 (2.0, 4.5)
CRP (mg/L)	1.9 (0.9, 4.4)
Fibrinogen (g/L)	3.6 ± 0.7

Data are presented as means ± SD, *n* (%) or median (lower IQR, upper IQR).

Maximum *n* = 1049.

a Note that there is overlap among these subgroups.

**Table 2:** Correlation coefficients between biomarkers at baseline (max n = 1032).^a^

	ABI < 1.4	NT-proBNP	hs-cTnT	GGT	TNF-α	IL-6	CRP	Fibrinogen	*g*
ABI < 1.4	1	−0.21***	−0.10**	−0.05	−0.07*	−0.12***	−0.13***	−0.18***	−0.18***
NT-proBNP		1	0.38***	−0.03	0.16***	0.18***	0.11***	0.21***	0.23***
hs-cTnT			1	0.03	0.19***	0.17***	−0.03	0.05	0.12***
GGT				1	0.08*	0.16***	0.24***	−0.06	0.16***
TNF-α					1	0.31***	0.12***	0.12***	0.43***
IL-6						1	0.42***	0.34***	0.75***
CRP							1	0.54***	0.80***
Fibrinogen								1	0.76***
*g*									1

*Pearson correlation test *p*-value < 0.05.

**Pearson correlation test *p*-value < 0.01.

***Pearson correlation test *p*-value <0.001.

a Missing data ranges from 8 to 39 data points.

A total of 205 first incident CV events (61 fatal/non-fatal MI, 38 angina, 53 stroke, 11 TIA, 24 coronary intervention and18 fatal IHD) occurred during the eight year follow-up period (19.5% of study population).

### 3.2 Individual biomarkers

The basic model had a C statistic of 0.722 (95% CI 0.681, 0.763) and was well-calibrated (Hosmer-Lemeshow *p* = 0.97). Individual biomarkers had much lower C statistics (95% CI) than the basic model; ABI 0.552 (0.486, 0.618), NT-proBNP 0.575 (0.511, 0.640); Troponin 0.601 (0.532, 0.669), Gamma-GT 0.554 (0.488, 0.621); g 0.580 (0.515, 0.645). Even when all five biomarkers were combined, the C statistic did not reach that of the basic model (0.642, 95% CI 0.577, 0.707).

Increased levels of circulating biomarkers were associated with an increased incidence of CV events over-and above the basic model (Table 3), but only the associations for NT-proBNP and hs-cTnT were statistically significant (*p*<0.05). The strongest association was observed for hs-cTnT (OR for 1 SD increase 1.35; 95% CI, 1.13, 1.61). A lower ABI was associated with a higher incidence of events (OR 0.86, 95% CI 0.73, 1.00).

**Table 3:** Adding biomarkers to the basic model (all columns refer to total population with complete case analysis, n = 989^a^, except for final column which gives c-statistics for sub-population with no CVD at baseline).

Predictors in the model, additional to conventional risk factors^b^	OR for a one SD increase in biomarker (95% CI)	C statistic (95% CI)	*p*-value for comparison with basic model	NR –event^c^(%)	NR –no event^c^ (%)	NRI	Goodness of fit (Hosmer-Lemeshow *p* value)	C statistic (95% CI) for sub-population with no baseline CVD
Basic model	–	0.722 (0.681, 0.763)					0.97	0.685 (0.623, 0.747)
ABI	0.86 (0.73, 1.00)	0.725 (0.684, 0.766)	0.44	−2.2	2.0	0.015	0.83	0.691 (0.628, 0.753)
NT-proBNP	1.23 (1.02, 1.49)	0.726 (0.685, 0.767)	0.39	−2.2	1.5	−0.007	0.81	0.684 (0.623, 0.745)
hs-cTnT	1.35 (1.13, 1.61)	0.732 (0.690, 0.774)	0.19	−1.6	2.2	0.006	0.09	0.685 (0.623, 0.747)
Gamma-GT	1.16 (0.98, 1.37)	0.726 (0.685, 0.766)	0.40	−2.7	1.1	−0.016	0.40	0.689 (0.626, 0.751)
*g*	1.07 (0.90, 1.27)	0.724 (0.683, 0.765)	0.29	0.5	1.2	0.018	0.90	0.693 (0.631, 0.755)
Top five models chosen (all-subsets regression)								
ABI, hs-cTnT, GGT	–	0.740 (0.699, 0.781)	0.04	−1.1	4.4	0.033	0.15	0.700 (0.637, 0.762)
ABI, hs-cTnT, GGT, proBNP	–	0.740 (0.699, 0.780)	0.06	−2.7	3.5	0.008	0.34	0.701 (0.640, 0.763)
hs-cTnT, GGT, proBNP	–	0.738 (0.697, 0.779)	0.07	−1.6	5.1	0.035	0.47	0.696 (0.634, 0.758)
ABI, hs-cTnT	–	0.735 (0.694, 0.776)	0.12	−3.2	5.4	0.021	0.35	0.695 (0.633, 0.756)
hs-cTnT, GGT	–	0.738 (0.697, 0.778)	0.06	−1.1	3.9	0.028	0.21	0.694 (0.632, 0.756)
Full model								
ABI, hs-cTnT, GGT, proBNP, *g*	–	0.740 (0.699, 0.781)	0.06	−1.6	5.2	0.036	0.39	0.706 (0.644, 0.767)

a A complete case analysis was carried out, n = 989 (n = 643, events = 83 for subpopulation with no CVD at baseline).

b Conventional risk factors: age, sex, smoking, atrial fibrillation, chronic kidney disease, arthritis, hypertension, BMI, sBP, total:HDL cholesterol, social status, baseline CVD status (MI, angina, TIA and stroke) and lipid lowering medication.

c n = 186 for event, n = 803 for no event.

Addition of each individual biomarker to the basic model increased the C statistic slightly, with the greatest increase seen for hs-cTnT (an increase of 0.010 from 0.722, 95% CI 0.681, 0.763 to 0.732, 95% CI 0.690, 0.774). Addition of individual biomarkers also improved the risk classification for participants who did not experience a CV event, although this generally resulted in poorer risk classification for participants who did experience a CV event (Table 3). The addition of hs-cTnT resulted in poorer risk classification by 1.6% for participants who experienced a CV event, but improved risk classification by 2.2% for those who did not. All the models were shown to be well-calibrated (Hosmer-Lemeshow *p* > 0.05).

### 3.3 Combinations of biomarkers

An all subsets regression was carried out and identified the top five models according to a pre-specified statistical criterion, after adjusting for conventional risk factors, from all possible combinations of biomarkers. All five models (Table 3) included hs-cTnT and none included the general inflammation factor, *g*. The best model selected using this method added ABI, hs-cTnT and GGT to the set of conventional CV risk factors. This model was well-calibrated and had a C statistic of 0.740 (95% CI 0.699, 0.781), an increase of 0.018 compared to the basic model (*p* = 0.04). The addition of the three biomarkers resulted in slightly poorer risk classification by 1.1% for participants who experienced a CV event, but improved risk classification by 4.4% for those who did not. The second best model was well-calibrated and showed the same increase in the C statistic as the top model, but the NR was poorer for participants who experienced a CV event (−2.7%s) and for those who did not (3.4%). For comparison, the full model including all biomarkers is also shown in Table 3. The C statistic showed the same increase as the top model. The addition of all biomarkers resulted in poorer risk classification by 1.6% for participants who experienced a CV event, but improved risk classification by 5.2% for those who did not.

A high proportion of participants had prevalent CVD at baseline and removing these subjects from analysis resulted in reduced statistical power (*n* = 643 subjects, *n* = 83 events) and a lower C statistic for the basic model (0.685, 95% CI 0.623, 0.747). Despite this, the increases in C statistic found on addition of the individual biomarkers to the basic model generally mirrored the increases seen in the full study population (Table 3). Also consistent with findings in the full study population, C statistics improved more for models including a combination of biomarkers than for those with individual biomarkers added (e.g. adding ABI, hs-cTnT and GGT to the basic model improved the C statistic from 0.685 to 0.700, *p* = 0.16).

## 4 Discussion

In older people with type 2 diabetes, a number of individual non-traditional biomarkers were associated with increased risk of incident CV events, independent of factors currently used to predict CVD. This included higher levels of hs-cTnT and NT-proBNP and a lower ABI. hs-cTnT appeared to be the most promising individual biomarker in terms of improving risk prediction over-and-above traditional risk factors incorporated in the QRISK2 score. Combinations of the non-traditional biomarkers further improved risk prediction compared with one strong biomarker alone, and biomarkers which on their own did not appear to add much predictive value still contributed to these models. The strongest predictive models included combinations of NT-proBNP, hs-cTnT, ABI and gamma GT, but did not include an inflammation factor representing four individual markers of systemic inflammation.

Cardiac troponin levels increase in response to clinical and subclinical myocardial ischaemia and is currently used to aid the diagnosis of myocardial infarction [17] while NT-proBNP is released by the heart in response to increased pressure on the ventricular wall with low levels used in clinical practice to rule out heart failure [16]. In previous studies on both the general population and in people with diabetes, hs-cTnT and NT-proBNP have been associated with the risk of CVD and may add predictive value independent of conventional risk factors [8], [9], [18], [19], [20]. A reduced ABI is used in the diagnosis of peripheral arterial disease, is a marker of generalized atherosclerosis and, in a meta-analysis capturing over 480,000 person years follow-up in the general population, improved CV risk prediction beyond the Framingham Risk Score [7]. More recently, two general population studies indicated that ABI had a small effect on CV risk and only improved risk prediction if the basic model was weak [21], [22], whilst evidence in diabetes has previously been lacking.

Previous evidence suggesting that inflammatory biomarkers, including CRP, IL-6, TNF-α and fibrinogen, may add predictive value independent of conventional risk factors in both general and diabetic populations is inconsistent [11], [20], [23], [24], [25], [26], [27]. Given that these four biomarkers are highly correlated, it has been suggested that they may best be combined into one general factor, which describes the overall inflammatory burden [12], [28], [29], [30]. This was the approach we chose for our study, but we found little evidence that the inflammatory factor was associated with incident events or predicted CV risk either individually or when combined with other biomarkers. Conversely, the liver function test, gamma GT, contributed to an increase in prediction when combined with other biomarkers, despite minimal evidence of a contribution when included on its own. GGT has previously been associated with CVD in two large general population studies [31], [32] and in people with type 2 diabetes [33], although it did not improve CV prediction beyond traditional risk factors [34]. Similarly, a recent general population cohort study of 2500 patients with acute coronary syndrome found that GGT was associated with increased risk of all-cause mortality but not cardiac mortality [35] and the PREVEND prospective cohort study suggested that adding GGT to conventional CV risk factors did not improve the prediction of first-ever CV events in the general population [36]. Our findings for gamma GT highlight the importance of avoiding pre-selecting biomarkers according to the statistical significance of their association with events prior to the inclusion of multiple biomarkers in a risk prediction model.

One of the strengths of the current study was the use of a basic model based on the risk score currently recommended for use in type 2 diabetes for risk prediction in the UK (the QRISK2 score, currently recommended by NICE clinical guidelines). However, precisely replicating the QRISK2 score in the ET2DS proved challenging. Family history of CVD was not available in the ET2DS and the SIMD was used as a measure of social status rather than the Townsend index, which is only applicable to England and Wales. The definition of CKD in QRISK2 is a clinical diagnosis of CKD, but the list of corresponding clinical codes is not readily available. A similar doctor-diagnosis definition of CKD created for the ET2DS only affected 1.7% of the cohort, much lower than anticipated in an elderly diabetic population [37], [38]. A new variable for CKD, based on an eGFR <60 ml/min (equivalent to Stage 3–5 CKD) identified 24.6% of the cohort and, as this was considered to be a more accurate definition of CKD, was used in subsequent analysis. Also QRISK2 excluded participants with previous CVD or taking statins and we were unable to use the statistical algorithm developed by QRISK2 as this was not made available to us.

Given the problems replicating QRISK2, and also in the knowledge that existing risk scores based on traditional vascular risk factors cannot be assumed to be more predictive and/or easier to apply in clinical practice than a score based on novel biomarkers alone, we considered C statistics for the biomarkers on their own (i.e. prior to the addition of traditional risk factors). However, this showed that biomarkers alone or in combination with each other were less predictive than traditional risk factors on their own (or combined with biomarkers), supporting our initial approach of adding biomarkers to the basic model based on the QRISK2 score. At 0.722, the C statistic for our basic model was similar in absolute value to those found by previous studies in type 2 diabetes, which used a variety of risk factor models and/or CV risk scores [9], [27], [39]. In our study, an even greater level of risk prediction was found when a combination of novel biomarkers was added to traditional risk factors, improving risk prediction beyond that seen with the addition of any single biomarker. Interestingly, an upper limit to model performance may be suggested by our finding of the same C statistic value for the two best models with different combinations of biomarkers and the model containing all the investigated biomarkers.

Because a very large proportion of the ET2DS had prevalent CVD at baseline or were taking lipid lowering medication (representing the situation in the target population of elderly people with type 2 diabetes) we included all subjects in our analyse, subsequently including prevalence of CVD and lipid lowering medication as additional covariates. Results of our sensitivity analysis in subjects free of CVD at baseline suggested that key findings were also similar in this more healthy sub-population. Our model therefore has the advantage of being potentially applicable to all people with type 2 diabetes, including those both with and without clinically-diagnosed CVD, all of whom could benefit from more accurate CV risk prediction. However, as power for our sensitivity analysis was limited, this findings needs to be confirmed in larger studies.

The size of improvements in the C statistic following the addition of various biomarkers was consistent with previous studies. Although the increases in C statistic were small, it should be noted that the C statistic can be insensitive when adding a new predictor to a model, even though such a predictor may make an independent and statistically significant contribution to the model [40]. This phenomenon is particularly noticeable when the baseline model includes strong predictors and has a large C statistic. In order to evaluate the clinical usefulness of our models, we also considered the NR as a measure of reclassification. This suggested that, in general, the risk classification improved after the addition of a biomarker for people who did not experience a CV event, but slightly worsened for people who did experience an event. Further large studies are needed to validate this conclusion and to ascertain whether any improvements are clinically significant.

Overall, this study benefited from the representativeness of the type 2 diabetes population, the relatively long term follow up for CV events and the thorough and systematic approach for assessing incident CV events which ensured loss to follow-up was minimal. The wide variety of biomarkers available allowed for the inclusion of a large panel of potential predictors, both individually and in combination. The study also has limitations. In addition to the insensitivity of the C statistic, the NR is dependent on the choice of risk thresholds. The continuous net reclassification index can be used to avoid this decision, but this is less clinically relevant. The NR should therefore be considered as a descriptive tool to demonstrate what would happen to risk scores in a clinical setting if the new model was used with the chosen risk categories (0–10%, 10–20%, >20%). Data were missing for some of the predictor variables and the complete case analysis performed can produce biased estimates or reduce statistical power. However, since missing data was less than 5%, and an analysis of subjects with missing data versus those without indicated that missing data rates did not depend on the outcome or key predictor variables, these risks were considered to be negligible.

In conclusion, our results suggest that in people with type 2 diabetes, a risk score based on a combination of both traditional and non-traditional (‘novel’) biomarkers may help identify patients who are at higher risk of cardiovascular mortality and morbidity and may be useful to stratify patients into those who are more or less likely to derive significant benefit from intensive preventive therapy or to avoid the unwanted side effects of unnecessary intervention. It has previously been suggested that it might be best to abandon estimates of individual risk in people with diabetes, and to treat all people with this condition as high risk [41]. Part of this argument is based on the lack of an accurate risk prediction tool, many of which have been developed in general population samples with only a small number of participants with diabetes, and which under estimate the risk of cardiovascular disease in people with diabetes. Our results suggest that not only can risk scores developed in the general population perform well in a diabetic population, but also that additional predictive ability can be achieved by adding a combination of non-traditional risk factor to an existing score based on traditional risk factors. Our results are particularly important in highlighting the incremental benefit that can be achieved by adding multiple novel risk factors to existing risk prediction models. However, before considering adopting any or all of the specific biomarkers identified in our ‘best’ model in clinical practice, our results need to be replicated in other cohorts, given that any model is likely to perform better in the population in which it is developed. If confirmed in future studies, consideration of the use of threshold values for each included biomarker may also help to increase the clinical utility of a risk score. We recommend that multiple traditional and non-traditional risk factors are considered both individually and in combination when identifying and testing clinically useful risk prediction scores for use in patients with diabetes, along with the equally important issue of the ease with which such variables can be measured in a primary or secondary care setting.

## Conflict of interest

The authors declared they do not have anything to disclose regarding conflict of interest with respect to this manuscript.

## Financial support

The sponsor for the ET2DS was the University Of Edinburgh. The study was funded by the Medical Research Council (UK) (Project Grant G0500877), the Chief Scientist Office of the Scottish Executive (Programme Support Grant CZQ/1/38), Pfizer plc. and Diabetes UK (Clinical Research Fellowship 10/0003985). The funders had no other role in the design, analysis or writing of this manuscript. CJW was supported in this work by NHS Lothian via the Edinburgh Clinical Trials Unit.

## Author contributions

JFP and MWJS conceived and designed the ET2DS and oversaw the acquisition and analysis of data. For the current paper, AHP, JFP and CW conceived the idea and designed the analysis, which was performed by AHP. AHP, JFP and CW wrote the paper. All authors contributed to data collection, interpretation of findings and preparation of the final manuscript, including commenting on the final draft. JFP and AHP are the guarantors of this work, and as such had full access to all the data in the study and take responsibility for the integrity of the data and the accuracy of the data analysis. The authors thank all patients and research staff involved in the ET2DS.
